# Homelessness Is Not Just a Housing Problem

**DOI:** 10.1371/journal.pmed.1000003

**Published:** 2008-12-23

**Authors:** The PLoS Medicine Editors

## Abstract

The*PLoS Medicine* Editors argue that political will and imaginative and collaborative solutions from across the spectrum of health and social care providers are needed to address the needs of homeless individuals.

Homelessness is about more than rooflessness. A home is not just a physical space: it provides roots, identity, security, a sense of belonging and a place of emotional wellbeing.” So says the United Kingdom charity Crisis [1]. This Editorial's title comes from another charity, Shelter [2], which breaks down the causes of homelessness into individual factors such as drug and alcohol misuse; lack of social support; family background, including family breakdown and disputes; and an institutional background, including being in prison, or increasingly and perhaps especially shamefully today, the armed services.

A systematic review published in this month's *PLoS Medicine* [3] looks at just one aspect of this complex issue—the prevalence of mental disorders in the homeless. The analysis shows that there is a substantial prevalence of mental disorders among homeless people in Western countries. Among prior studies meeting criteria for consideration, the prevalence of alcohol dependency ranged from 8.1%–58.5%, and drug dependence ranged from 4.5%–54.2%. For psychotic illnesses, the prevalence ranged from 2.8%–42.3%, with similar findings for major depression. What these figures don't show, of course, is whether mental illnesses are the cause or consequence of homelessness—or, as seems more likely, whether these conditions are mutually contributory and their causes interrelated. What the results do reinforce is that homeless individuals have substantial and complex health needs. Helen Herrman, professor of psychiatry at the University of Melbourne, Australia, notes in her Perspective on Fazel and colleagues' paper [4] that these needs are not well documented: “It is a stark finding that despite all that has been written about mental illness among homeless people, and all the speculation about the origin of the problem, the authors deemed only 29 studies relevant to their analysis.” And of course this paper only includes individuals in Western countries; worldwide, the health needs of an even more vulnerable group of homeless people—refugees and persons displaced by conflict—are even more challenging.

Why are papers such as the one by Fazel and colleagues, which document these health needs, so important? If the health needs of groups are understudied and underrepresented in the medical literature, it is likely that their health needs are not being addressed equably by society as a whole. A case report from 1993 [5] of two homeless men whose condition improved substantially when they were diagnosed with schizophrenia and treated with antipsychotics illustrates that homeless individuals, simply by the fact of being homeless, may fail to get the treatment that someone in a housed environment would receive. As the authors of the case report say, these individuals “have tenuous social supports which collapse when they develop psychoses. Instead of being brought to psychiatric attention and receiving appropriate treatment at these times of crisis, they may drift into hostels for the homeless. Here they remain without attracting psychiatric attention, because hostel staff… have learnt to tolerate a level of disturbance which relatives, neighbours or landlords would not.” [5]. These cases neatly illustrate the complexity and interrelatedness of social and health support systems that are required to keep an individual a healthy member of society.

Perhaps one reason why it is hard to provide appropriate care for the homeless is that they are of course not a homogenous population, nor are they easy to count. Many people would consider the homeless to include only those who sleep on the streets, but considering only these people vastly underestimates the size of the problem. The statistics in England alone are an illustration of the difficulties of counting this population. UK government figures suggest that 500 people sleep rough on any one night. However, according to Crisis, 120,000 households in England were officially recognized as being homeless under the legal definition in 2006, but there are in addition around 400,000 “hidden homeless” adults at any one time, who may or may not have applied to the appropriate authorities for homeless status, and who are thus not entitled to any accommodation and not counted.

To directly translate these figures into effects on health is hard, again because of the relative lack of data. However, one substantial study on mortality of men in hostels in Toronto [6] showed increased mortality rate ratios among all age groups compared with men in the general population, ranging from 8.3 times higher for men aged 18–24 years, to 2.3 times higher for men aged 45–64 years. A study of homeless women, also in Toronto [7], confirmed the higher relative increases in deaths in a younger age group: homeless women 18–44 years of age were 10 times more likely to die than women in the general population of Toronto.

Practically, why is there such a higher risk of death in the homeless? Again the answers are not simple. Hwang [6] noted that homeless people are more exposed to the elements, but of course there are other, much harder to modify factors, some which may be part of the cause and not the consequence of homelessness. Apart from the major mental disorders noted in the paper by Fazel et al., other health issues that are overrepresented or impose a substantial burden on the homeless are violence [8], including traumatic head injury [9]; pregnancy (especially among teenage homeless girls) [10]; HIV [11]; and smoking-related illnesses (the tobacco industry in the past specifically has targeted the homeless [12]. What this by no means exclusive list serves to illustrate is the complex health needs of the homeless—and that no one approach can possibly serve for all.

As one of the most visible and intractable problems in modern society, homelessness reveals and magnifies the structural shortcomings of our current social fabric. The global financial crisis has reminded everyone of how precarious a seemingly secure lifestyle can be, and that established protections for the most fortunate in society are simply not inclusive enough, especially when times get tough. Addressing the problem of homelessness therefore requires not only a proper understanding of its size, and of its association with ill health (as examined by Fazel and colleagues), but also attention to an often overlooked basic rights—equal access to health care. The Toronto study of mortality in homeless men had one positive finding (relatively speaking) in that the mortality rate of these men was much lower than in comparable populations in the United States. The authors speculate on the reasons for this and highlight one issue: that in Canada there is much better access to health care than in the US. However, even access to care is not a guarantee that the most needy will avail themselves of it: in the UK, a country with universal health care, homeless people are 40 times less likely to be registered with a general practitioner than the population as a whole.

Imaginative and collaborative solutions from across the whole spectrum of health and social care providers are needed—for example, targeted health care interventions such as mobile clinics and community outreach. But charities such as Crisis and Shelter are already warning that the risk of homelessness for many people is closer than we have previously assumed and now more than ever, homeless people can no longer be considered outside of society. Political will at the highest level is needed to put them back into the mainstream political and therefore health and social agenda.

## References

[pmed-1000003-b001] Crisis (2008). About homelessness. http://www.crisis.org.uk/policywatch/pages/about_homelessness.html.

[pmed-1000003-b002] Shelter (2008). What causes homelessness. http://england.shelter.org.uk/housing_issues/homelessness/What_causes_homelessness#0.

[pmed-1000003-b003] Fazel S, Khosla V, Doll H, Geddes J (2008). The prevalence of mental disorders among the homeless in Western countries: Systematic review and meta-regression analysis. PLoS Med.

[pmed-1000003-b004] Herrman H (2008). Mental disorders among homeless people in Western countries. PLoS Med.

[pmed-1000003-b005] Marshall M, Sharpe M (1993). Untreated schizophrenia in hostels for the homeless: A cause for concern. Psychiatr Bull.

[pmed-1000003-b006] Hwang SW (2000). Mortality among men using homeless shelters in Toronto, Ontario. JAMA.

[pmed-1000003-b007] Cheung AM, Hwang SW (2004). Risk of death among homeless women: A cohort study and review of the literature. CMAJ.

[pmed-1000003-b008] Kushel MB, Evans JL, Perry S, Robertson MJ, Moss AR (2003). No door to lock: Victimization among homeless and marginally housed persons. Arch Intern Med.

[pmed-1000003-b009] Hwang SW, Colantonio A, Chiu S, Tolomiczenko G, Kiss A (2008). The effect of traumatic brain injury on the health of homeless people. CMAJ.

[pmed-1000003-b010] Thompson SJ, Bender KA, Lewis CM, Watkins R (2008). Runaway and pregnant: Risk factors associated with pregnancy in a national sample of runaway/homeless female adolescents. J Adolesc Health.

[pmed-1000003-b011] Zetola NM, Grijalva CG, Gertler S, Hare CB, Kaplan B (2008). Simplifying consent for HIV testing is associated with an increase in HIV testing and case detection in highest risk groups, San Francisco January 2003–June 2007. PLoS ONE.

[pmed-1000003-b012] Apollonio DE, Malone RE (2005). Marketing to the marginalised: Tobacco industry targeting of the homeless and mentally ill. Tob Control.

